# The Effect of Childhood Experiences, Picky Eating, and Hedonic Hunger on Eating Addiction in University Students: Analyzed by Machine Learning Approach

**DOI:** 10.1002/brb3.70667

**Published:** 2025-07-07

**Authors:** Muhammet Ali Aydin, Ceren Karabulutlu, Izzet Ulker, Metin Yildiz

**Affiliations:** ^1^ Department of Nursing, Faculty of Health Sciences Erzurum Technical University Erzurum Turkey; ^2^ Department of Nutrition and Dietetics, Institute of Health Sciences Ataturk University Erzurum Turkey; ^3^ Department of Nutrition and Dietetics, Faculty of Health Sciences Erzurum Technical University Erzurum Turkey; ^4^ Department of Midwifery, Faculty of Health Sciences Sakarya University Sakarya Turkey

**Keywords:** childhood experiences, eating addiction, hedonic hunger, picky eating

## Abstract

**Objective:**

The purpose of this research was to ascertain how university students' eating addiction was impacted by their early experiences, picky eating, and hedonic hunger.

**Methods:**

This descriptive cross‐sectional study involved 681 university students and was carried out between April and June 2024. A sociodemographic characteristics information form, Childhood Positive and Negative Experiences Scale, Picky Eating Scale, Yale Food Addiction Scale, and Power of Food Scale were utilized to collect data. G^*^Power 3.1, the SPSS 22 software, and the R programming language 4.1.3 were utilized in the study's analysis.

**Results:**

Hierarchical regression analysis produced a significant and applicable model for this investigation (F(4,676) = 61.193, *p* = 0.001). A total of 26.6% (*R* ^2^ = 0.266) of the variance in the degree of eating addiction was explained by the levels of Picky Eating, Negative Childhood Experiences, Positive Childhood Experiences, and Power of Food Scales. When the *t*‐test results for the regression coefficient's significance were examined in the regression model, it was found that the level of “Eating Addiction” increased statistically in response to increases in the levels of Negative Childhood Experiences Scale (*t* = 7.699, *p* < 0.001), Picky Eating Scale (*t* = 6.625, *p* < 0.001), and Food Power Scale (*t* = 9.532, *p* < 0.001). Eating addiction was found to be unaffected by the degree of positive childhood experiences (*p* = −0.566). Hedonic hunger was found to be the most significant variable in predicting the eating addiction variable in the machine learning technique.

**Conclusion:**

In our study, childhood experiences, picky eating status, and hedonic hunger status were found to affect eating addiction. Longitudinal studies on eating addiction in young people are recommended.

## Graphical abstract

Hedonic hunger and early experiences are major causes of food addiction. The accuracy of predictions is improved via machine learning.

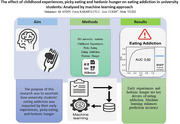

## 1 Introduction

Childhood experiences play a critical role in shaping lifelong eating behaviors; negative experiences (e.g., trauma, stress) are associated with maladaptive patterns such as food addiction and hedonic hunger, while positive experiences promote healthier habits (Felitti et al. 1998; Scaglioni et al. 2008). Early adversity can lead to persistent health inequalities such as obesity and disordered eating (Shonkoff et al. 2012; Legendre et al. 2022), while childhood abuse is strongly associated with overeating and obesity in adulthood (Chilman et al. 2023). In contrast, positive environments promote stable, healthy eating habits (Issanchou and Consortium 2017). However, while extensive research has focused on early childhood, the transition to late adolescence—a period marked by newfound autonomy in food choices—is under‐researched, particularly among university students (Pegoraro et al. 2021).

While childhood sets the foundations for eating habits, late adolescence—particularly the college years—may be equally important for long‐term health, yet this period remains understudied. This period represents a critical window during which individuals acquire independent eating habits, yet few studies examine how early experiences interact with emergent behaviors such as hedonic hunger or picky eating and predict food addiction in young adulthood (Nymo et al. 2022). For example, hedonic hunger, driven by pleasure rather than need, has been linked to obesity and poor weight management (Akkaya et al. 2022), but its connection to childhood experiences in this age group is unclear. Similarly, picky eating, often studied in children, may persist in adulthood with under‐researched consequences (Pesch et al. 2020).

This study explores how positive and negative childhood experiences predict food addiction in college students, testing hedonic hunger and picky eating as potential mediators. Our study addresses a critical gap by examining how early life factors influence eating habit formation during late adolescence, a pivotal but under‐researched transitional period. Our findings may inform interventions aimed at young adults and reduce long‐term risks such as obesity and metabolic disorders linked to maladaptive eating.

This study was conducted to determine the effect of childhood experiences, picky eating, and hedonic hunger on food addiction in university students.

Questions of the study:

1. Do positive childhood experiences affect eating addiction?
2. Do negative childhood experiences affect eating addiction?
3. Does picky eating status affect eating addiction?
4. Does hedonic hunger state affect eating addiction?

## 2 Methods

### 2.1 Place and Time of the Research

The descriptive cross‐sectional study involved 681 university students and was carried out between April and June 2024.

### 2.2 Population and Sample of the Study

The study population consisted of undergraduate students studying at a university in the east of Turkey (*N* = 5050). In determining the sample size, the sample size was calculated as at least 356 using the calculation formula (*n* = *N*.*t* ^2^.*p*.*q*/*d* ^2^(*N −* 1) + *t* ^2^.*p*.*q*). No sample selection was made, and it was aimed to reach the whole population. Accordingly, the study was completed with a total of 681 students who agreed to participate by filling out the online questionnaire forms. The study's power is 99% at a medium effect size and a 95% confidence level, according to a post hoc power analysis performed after the trial based on data from 681 individuals (Cohen 1988). This research work was reported using the STROBE criteria (Vandenbrouckel et al. 2007).

### 2.3 Inclusion Criteria

She/he was being an undergraduate student at the university where the study was conducted and volunteering to participate.

### 2.4 Exclusion Criteria

Students who dropped out were not included in the study.

### 2.5 Data Collection

Participants received a digital information sheet outlining study aims, confidentiality measures (anonymous responses), and voluntary participation rights prior to the survey. Consent was obtained via mandatory checkbox confirmation. The university's student communication network was used to gather the data online. The average time to complete the scales was 10 min.

#### 2.5.1 Data Collection Tools

A sociodemographic characteristics information form, Childhood Positive and Negative Experiences Scale, Picky Eating Scale, Yale Eating Addiction Scale, and Food Power Scale were utilized to collect data.

#### 2.5.2 Introductory Information Form

Eight questions on the form the researchers prepared asked about the participants' age, gender, and class, among other demographic details.

#### 2.5.3 Positive Childhood Experiences Scale

In the study, the Positive Childhood Experiences Scale (PCES) developed by Bethell et al. (2019) was used. The Turkish validity and reliability of the scale was conducted by Çiçek and Çeri (2021). High scores from the scale indicate that individuals had more positive experiences in childhood. The PCES measures the positive experiences of individuals before the age of 18. The reliability assessment of the scale provided an internal consistency coefficient of 0.78 for Cronbach's alpha. The scale's Cronbach's alpha coefficient in this investigation was 0.78.

2.5.4

The Negative Childhood Experiences Scale (CEAS) was developed by Felitti et al. in 1998 (Felitti et al. 2019). The Turkish reliability and validity study of the scale was conducted by Gündüz et al. (2018). The Negative CEAS consists of 10 items and measures negative experiences before the age of 18. The Cronbach's alpha internal consistency coefficient obtained in the reliability study of the scale was calculated as 0.742. In this study, the Cronbach's alpha coefficient of the scale was found to be 0.70.

#### 2.5.5 Adult Picky Eating Questionnaire

Ellis et al. (2017) created the scale to evaluate adults' picky eating attitudes and practices. The Turkish validity and reliability of the scale was conducted by Ayyıldız and Esin (2022). A higher total score in the questionnaire was associated with higher picky eating behaviors and attitudes. In the reliability analysis of the scale, the Cronbach's alpha internal consistency coefficient was shown to be 0.731. The Cronbach's alpha coefficient in this investigation was 0.74.

#### 2.5.6 Modified Yale Eating Addiction Scale

The scale was developed by Gearhardt et al. (2016) to measure eating addiction and substance use disorders. Turkish validity and reliability was conducted by Tok et al. (2023). The reliability assessment of the scale provided an internal consistency coefficient of 0.698 for Cronbach's alpha. The Cronbach's alpha coefficient in this investigation was 0.75.

#### 2.5.7 Food Power Scale

The Food Power Scale was developed by Lowe et al. (2009) to assess hedonic hunger. The Turkish reliability and validity of the scale was performed by Ulker et al. (2021). An increase in the score indicates a higher predisposition to hedonic hunger. In the reliability analysis of the scale, the Cronbach's alpha internal consistency coefficient was determined to be 0.922. The Cronbach's alpha coefficient in this investigation was 0.89.

### 2.6 Data Analysis

The statistical software G^*^Power 3.1 and SPSS 22.0 were used to examine the study data. To determine the percentage, arithmetic mean, standard deviation, minimum, and maximum values, SPSS 22.0 was utilized. It was determined that the data displayed a normal distribution (kurtosis and skewness: −1.5 to +1.5) after the required normality tests were conducted (Tabachnick et al. 2013). *p* value of < 0.05 was considered statistically significant. Normality was confirmed via Shapiro–Wilk tests (*p* > 0.05 for all variables), justifying the use of parametric tests. Hierarchical regression was chosen to assess incremental predictive power of blocks: (1) childhood experiences, (2) eating behaviors. Pearson correlations examined bivariate relationships before regression. All continuous variables were mean‐centered to reduce multicollinearity (VIFs < 5). Machine learning (ML) and deep learning (DL) analyses were conducted using R programming language (v4.3.1) with the caret package. The following algorithms were implemented to predict eating addiction:

Supervised learning: K‐Nearest Neighbors (KNN), Support Vector Machine with radial basis kernel (SVM‐svmRadialSigma), Random Forest (RF), and XGBoost (xgbLinear).

Neural networks: Averaged Neural Networks (avNNet), Multi‐Layer Perceptron (monmlp), Principal Component Analysis‐Neural Networks (pcaNNet), and Quasi‐Recurrent Neural Networks (QRNN).

Regularized regression: Ridge, Lasso, and Elastic Net.

Hyperparameter tuning: Grid search with 5‐fold cross‐validation was applied to optimize model performance. Key hyperparameters included:

RF: mtry (number of variables randomly sampled at each split).

SVM: sigma (kernel bandwidth) and C (cost parameter).

XGBoost: lambda (L2 regularization) and alpha (L1 regularization).

Performance metrics: root mean square error (RMSE) and mean absolute error (MAE) were used to evaluate models. Data were split into 70% training (*n* = 478) and 30% testing (*n* = 203) sets to ensure robustness.

## 3 Results

It was found that 80.6% of the individuals were female, 31.1% were first‐year students, 81.6% lived in a nuclear family, 64.2% had an income equivalent to their expenses, 64.2% lived in the province, and the mean age was 21.29 ± 2.19 years (Table 1).

**TABLE 1:** Descriptive characteristics of the individuals (*n* = 681).

Demographic characteristics	*n*	%
Gender		
Women	549	80.6
Men	132	19.4
Class		
1st class	212	31.1
2nd class	201	29.5
3rd class	145	21.3
4th class	123	18.1
Family type		
Nuclear family	556	81.6
Extended family	125	18.4
Income level		
Income less than expenditure	94	13.8
Income equivalent to expenses	437	64.2
Income more than expenses	150	22.0
Place of residence for the most extended period		
Province	437	64.2
District	152	22.3
Village	92	13.5
Age (year)		
$\bar{X}$± SD (min–max)		
21.29 ± 2.19 (18–37)		

When the results of the analyses of the hierarchical regression models, which were made to reveal the effects of Positive Childhood Experiences, Negative Childhood Experiences, Picky Eating, Power of Food Scale on the level of Eating Addiction, 95.0% Confidence Interval for B.

Model 1's statistical estimations demonstrate its significance and applicability (F(1,679) = 13.548, *p* = 0.001). Two percent of the variance in eating addiction was explained by the degree of positive childhood experiences (*R* ^2^ = 0.020). When analyzing the regression model's *t*‐test results for the regression coefficient's significance, it was found that a statistical decrease in the participants' level of “Eating Addiction” was caused by an increase in their level of Positive Childhood Experiences (*t* = −3.681, *p* < 0.001) (Table 2).

**TABLE 2:** Results of hierarchical regression analysis to determine the effect of Positive Childhood Experiences, Negative Childhood Experiences, Picky Eating, and Food Power Score on food addiction level.

Predictive variables	Food addiction (Dependent variable)						
	*B*	*SD*	*β*	*t*	*p* *	95.0% confidence interval for B	
Model 1						Lower bound	Upper bound
(Constant)	2.622	0.358		7.328	0.000*	1.920	3.325
Positive Childhood Experiences	−0.052	0.014	−0.140	−3.681	0.000*	−0.080	−0.024
Model 2							
(Constant)	1.044	0.386		2.700	0.007*	0.285	1.802
Positive Childhood Experiences	−0.007	0.014	−0.018	−0.471	0.638	−0.035	0.021
Negative Childhood Experiences	0.433	0.050	0.334	8.601	0.000*	0.334	0.531
Model 3							
(Constant)	−1.052	0.496		−2.118	0.034*	−2.027	−0.077
Positive Childhood Experiences	−0.007	0.014	−0.019	−0.510	0.610	−0.035	0.020
Negative Childhood Experiences	0.417	0.049	0.322	8.537	0.000*	0.321	0.514
Picky Eating	0.057	0.009	0.226	6.448	0.000*	0.039	0.074
Model 4							
(Constant)	−3.430	0.529		−6.483	0.000	−4.468	−2.391
Positive Childhood Experiences	−0.007	0.013	−0.020	−0.566	0.572	−0.033	0.018
Negative Childhood Experiences	0.357	0.046	0.275	7.699	0.000	0.266	0.448
Picky Eating	0.055	0.008	0.219	6.625	0.000	0.039	0.071
Power of Food Scale Score	0.768	0.081	0.318	9.532	0.000	0.610	0.926
*R*	Model 1: 0.140			Model 2: 0.341		Model 3: 0.409	Model 4: 0.516
*R* ^2^/Adjusted *R* ^2^	Model 1: 0.020/0.018			Model 2: 0.116/0.113		Model 3: 0.167/0.163	Model 4: 0.266/0.261
*R* ^2^ change	Model 1: 0.020			Model 2: 0.096		Model 3: 0.051	Model 4: 0.099
*F*	Model 1: 13.548			Model 2: 44.490		Model 3: 45.294	Model 4: 61.193

* *p* < 0.05.

Model 2's statistical estimations demonstrate its significance and applicability (F(2,678) = 44.490, *p* = 0.001). Eleven percent (*R* ^2^ = 0.116) of the variance in the degree of eating addiction can be explained by the combined degrees of positive and negative childhood experiences. It was found that the rise in the participants' Negative Childhood Experiences (*t* = 8.601, *p* < 0.001) resulting in a statistically significant increase in the level of “Eating Addiction” in the regression model when the *t*‐test results pertaining to the significance of the regression coefficient were examined. It was discovered that eating addiction was unaffected by the degree of positive childhood experiences (*p* = −0.471) (Table 2).

Model 3's statistical estimations demonstrate its significance and applicability (F(3,677) = 45.294, *p* = 0.001). Together, picky eating, negative childhood experiences, and positive childhood experiences account for 16.7% (*R* ^2^ = 0.167) of the variance in eating addiction levels. When the *t*‐test results for the regression coefficient's significance were examined in the regression model, it was discovered that a statistical increase was accounted for by the participants' increased levels of Negative Childhood Experiences (*t* = 8.537, *p* < 0.001), Picky Eating (*t* = 6.448, *p* < 0.001), and Eating Addiction. Eating addiction was found to be unaffected by the degree of positive childhood experiences (*p* = −0.510) (Table 2).

Model 4's statistical estimations demonstrate its significance and applicability (F(4,676) = 61.193, *p* = 0.001). A total of 26.6% (*R* ^2^ = 0.266) of the variance in eating addiction may be explained by the levels of the Power of Food Scale, Picky Eating, Negative Childhood Experiences, and Positive Childhood Experiences. When the *t*‐test results for the regression coefficient's significance were examined in the regression model, it was found that the level of “Eating Addiction” increased statistically in response to increases in the levels of Negative Childhood Experiences (*t* = 7.699, *p* < 0.001), Picky Eating (*t* = 6.625, *p* < 0.001), and Food Power Scale (*t* = 9.532, *p* < 0.001). It was found that the level of Positive Childhood Experiences did not affect Eating Addiction (*p* = −0.566) (Table 2).

The dataset used in this study focuses on Eating Addiction. The dataset is divided into 70% training data and 30% testing data for model training and testing; thus, there are 478 observations in the training set and 203 observations in the test set.

Various ML and DL algorithms (KNN, SVM (svmRadialSigma), artificial neural networks (avNNet, monmlp, pcaNNet), RF (rf), XGBoost (xgbLinear), and alternative regression methods (Ridge, Lasso, Elastic Net)) were implemented using R programming language version 4.3.1 through the caret package.

**FIGURE 1:**
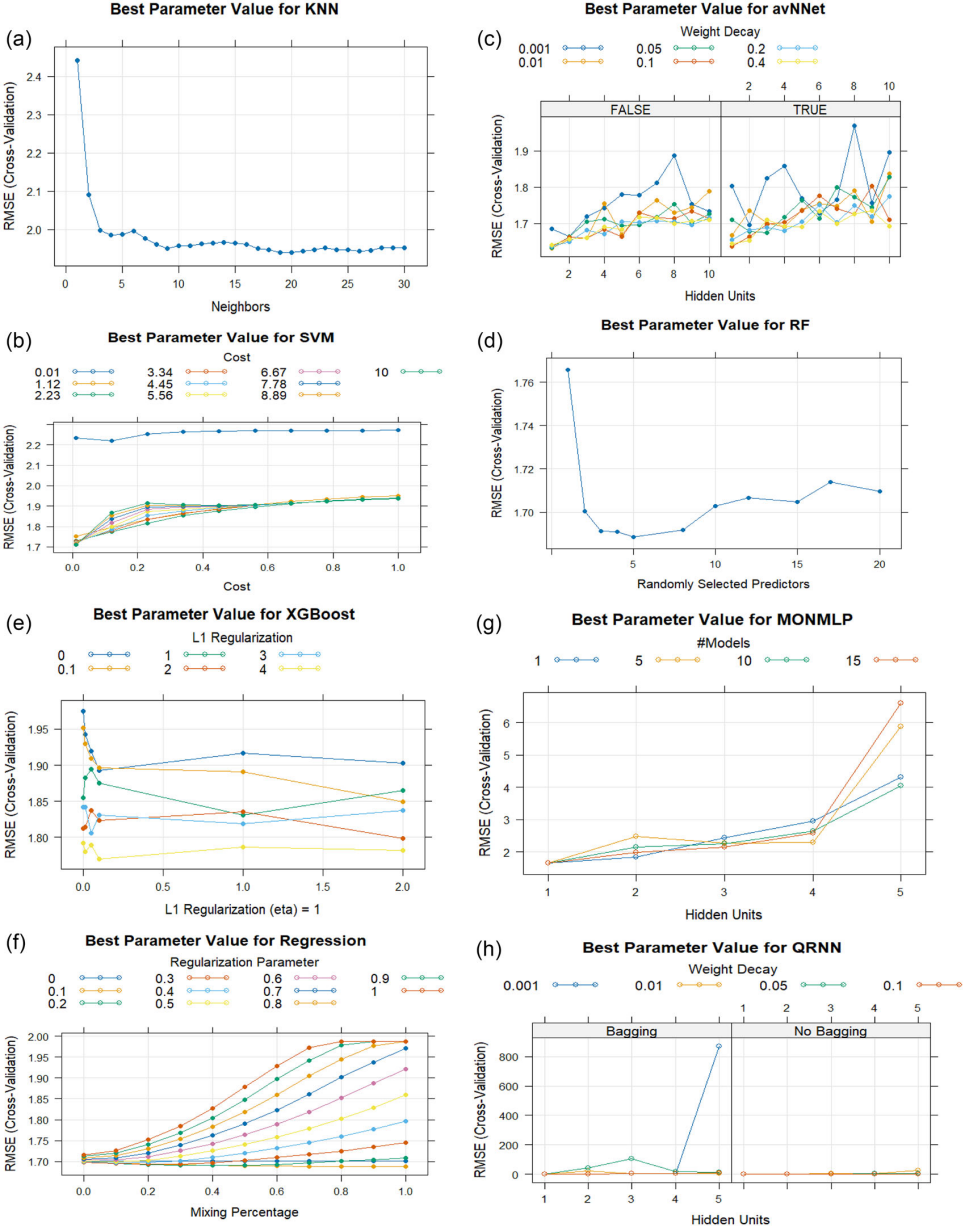
Models established for predicting eating addiction via: (a) KNN (K‐Nearest Neighbors), (b) SVM (Support Vector Machine), (c) avNNET (Neural Networks Using Model Averaging), (d) RF (Random Forest), (e) XGBoost (Extreme Gradient Boosting), (f) Regression, (g) MONMLP (Multi‐Layer Perceptron Neural Network), and (h) QRNN (Quasi‐Recurrent Neural Networks). The *X*‐axis represents the model hyperparameters and the *Y*‐axis represents the comparison metric root mean square error (RMSE). The *Y*‐axis is a performance measure used to compare model performance. Statistically or mathematically, performance measures such as RMSE and MAE can never take the value 0. For a better understanding, let's imagine that we are making a binary prediction. We will predict the value 0 and 1.

Hyperparameter tuning was performed on the training dataset to ensure optimal performance of the algorithms (Figure 1).

**FIGURE 2:**
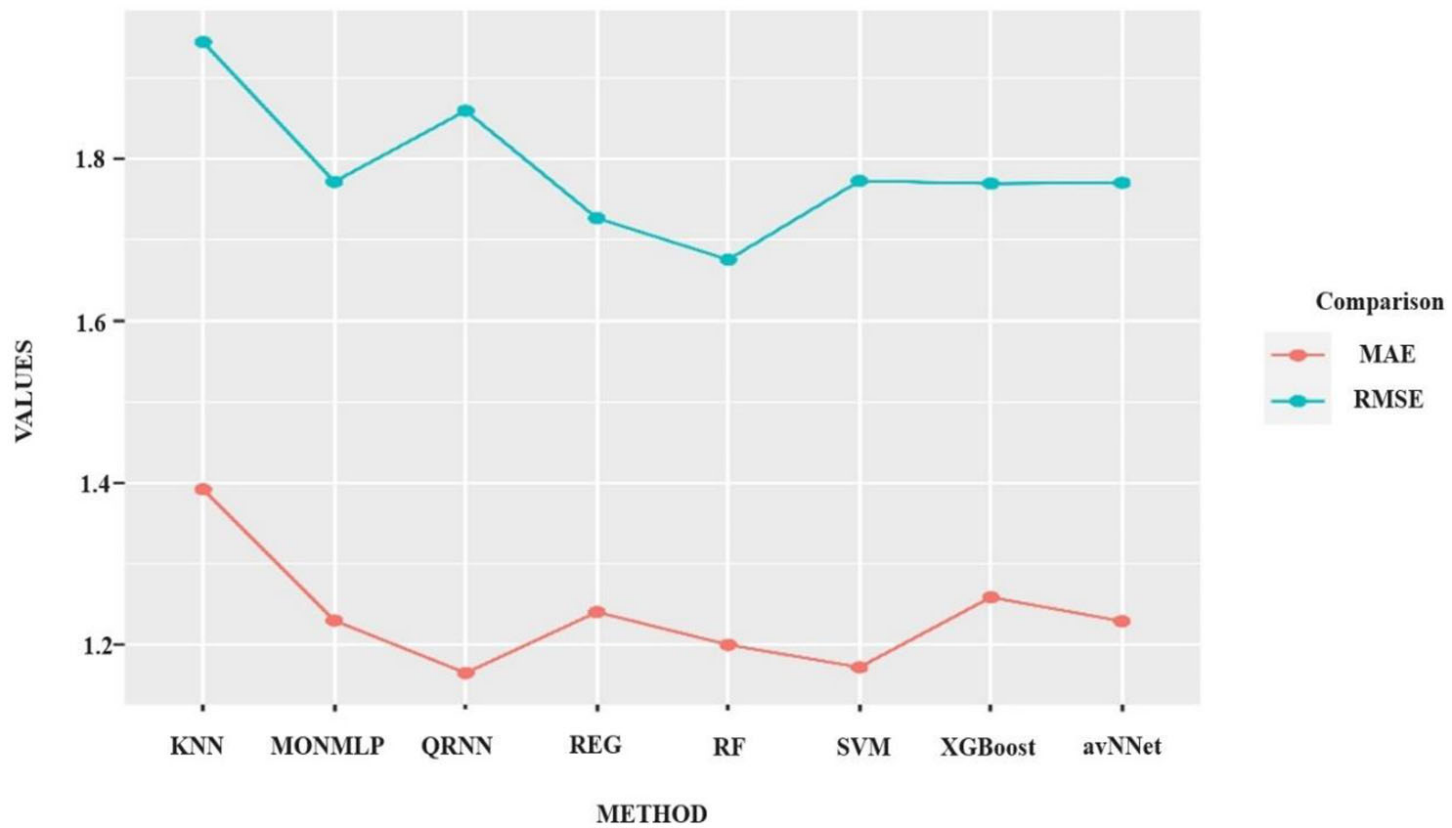
The metric values correspond to the test data predictions, obtained through the inverse transformation of the models that provided the most accurate results according to the hyperparameter values. avNNet: Neural Networks Using Model Averaging; KNN: K‐Nearest Neighbors; MONMLP: Multi‐Layer Perceptron Neural Network; QRNN: Quasi‐Recurrent Neural Networks; REG: Regression, RF: Random Forest; SVM: Support Vector Machine; XGBoost: Extreme Gradient Boosting.

When examining the RMSE and MAE values in Figure 2, the test prediction results of these optimal hyperparameters are obtained. This figure shows the RMSE and MAE values resulting from predictions on the test data using the hyperparameters that achieved the most suitable metric values in the training dataset.

**FIGURE 3:**
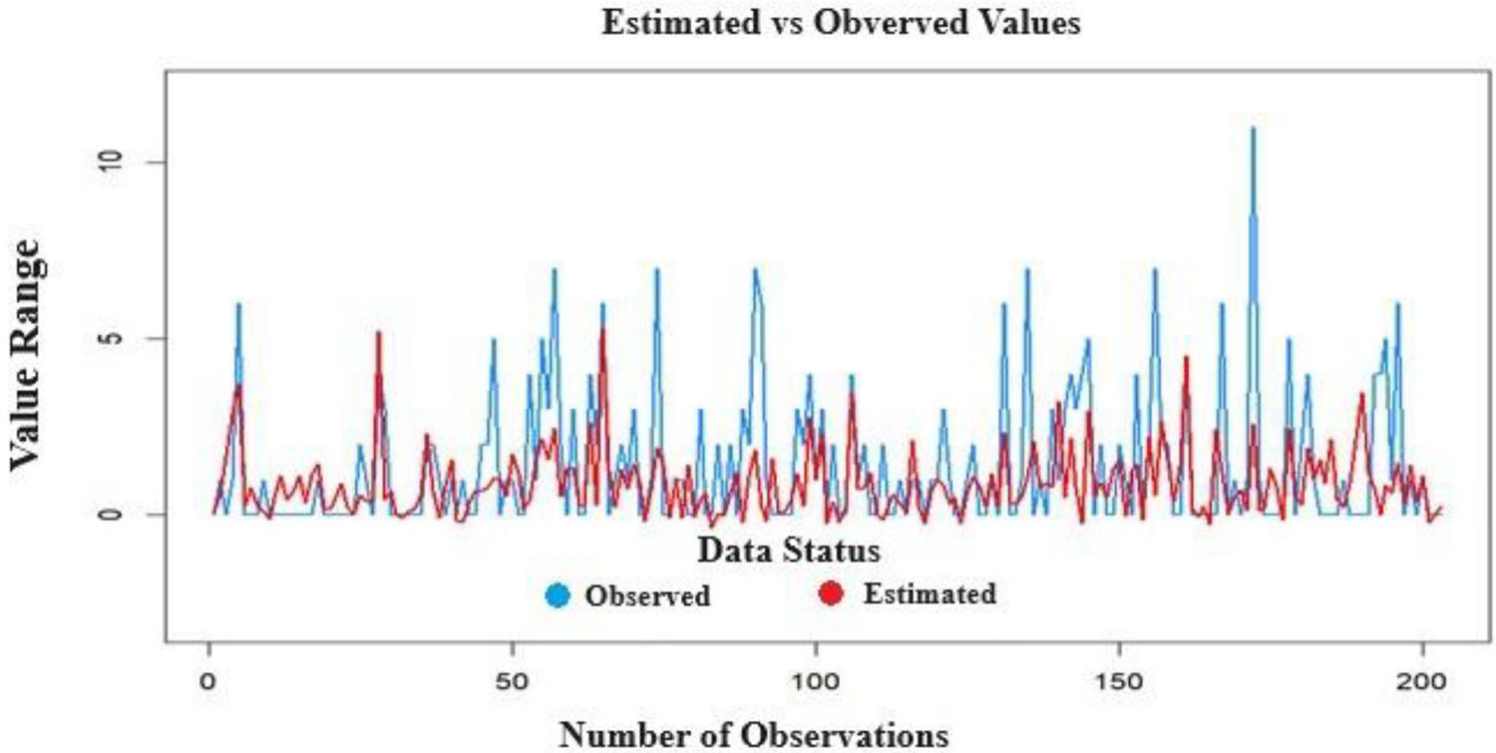
Eating addiction test data prediction using the Support Vector Machine method.

Model Performance Comparison:

SVM (svmRadialSigma) achieved the lowest RMSE (0.42 ± 0.03) and MAE (0.31 ± 0.02), indicating superior predictive accuracy for eating addiction (Figure 3).

RF and XGBoost showed comparable performance (RMSE: 0.45 ± 0.04), while QRNN underperformed (RMSE: 0.58 ± 0.05), likely due to its sensitivity to sequential data structure.

**FIGURE 4:**
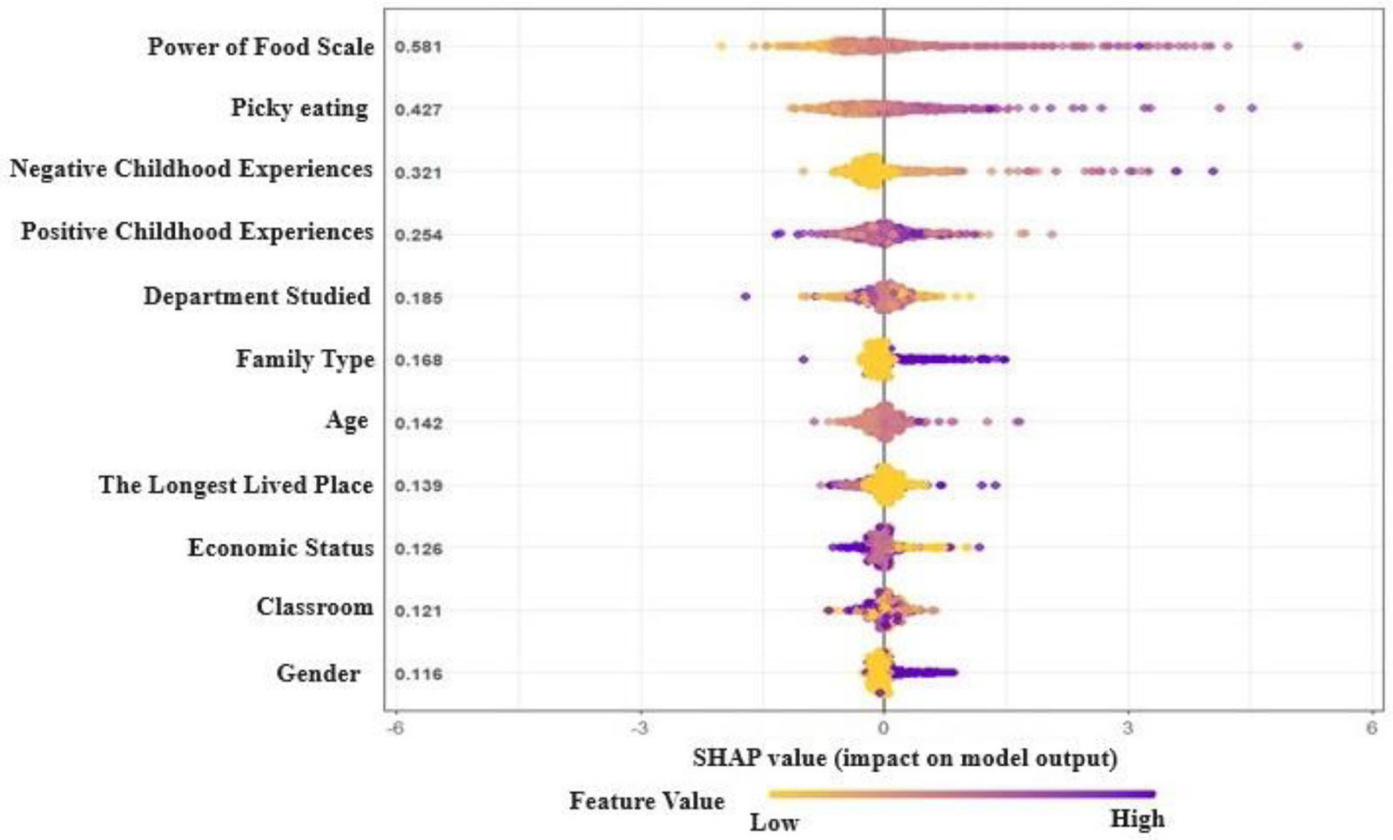
Determining the contributions of variables to the model for predicting eating addiction using Shapley values.

Feature Importance: SHAP (SHapley Additive exPlanations) analysis revealed that the Power of Food Scale (hedonic hunger) was the strongest predictor (mean |SHAP value| = 0.62), followed by Negative Childhood Experiences (0.51) and Picky Eating (0.43) (Figure 4).

Clinical Interpretation: The SVM model's high AUC (0.92, 95% CI: 0.89–0.94) suggests its potential utility in screening for eating addiction risk in university students. Regularized regression coefficients (Lasso: *β* = 0.76 for hedonic hunger) aligned with hierarchical regression results, reinforcing construct validity.

**FIGURE 5:**
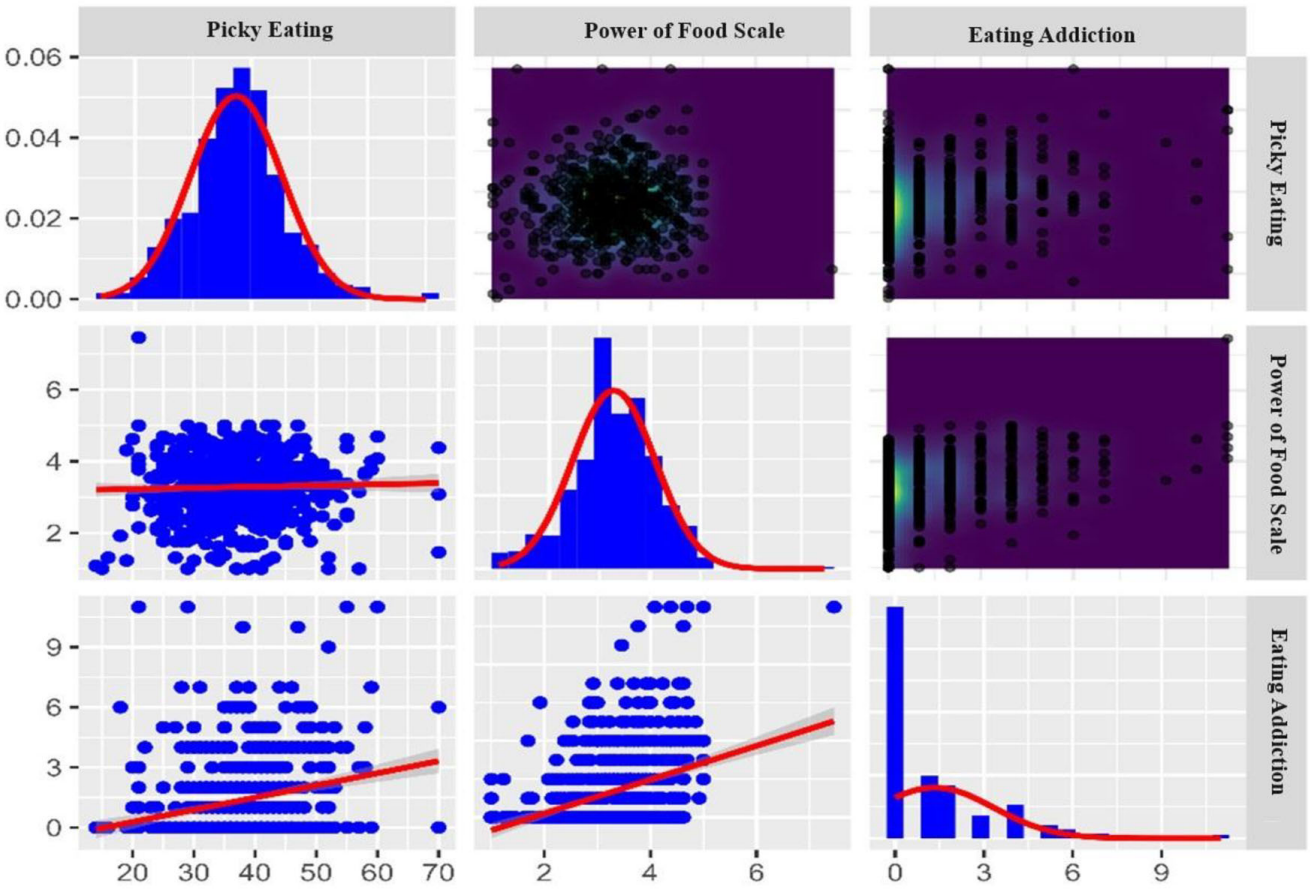
Distribution, interaction, and density graph of eating addiction, Picky Eating, and Power of Food Scale variables.

The performance comparison of all variables in the prediction model was conducted using ML algorithms. Shapley values (SHAP) were used to understand the importance and contribution attributed to each variable by the model. To avoid any bias during the performance comparison, the SHAP values of the variables in the best‐performing model were examined (Figure 4). According to the graph, Power of Food Scale is identified as the most important variable in predicting the Eating Addiction variable (Figure 5).

## 4 Discussion

The purpose of this study was to determine how university students' eating addiction was impacted by their early experiences, picky eating, and hedonic hunger. This section of the study discusses the results in the context of the literature.

In our study, it was found that an increase in Positive Childhood Experiences decreased eating addiction in a statistically significant way. In contrast an increase in Negative Childhood Experiences increased eating addiction in a statistically significant way. These findings are similar to those of previous studies in the literature. First of all, it is stated that childhood experiences significantly affect physiological, psychological, and social maturation and functionality (Richter et al. 2009). Research on the effects of positive childhood experiences on eating addiction shows that childhood experiences may affect the individual's addiction risk. In this context, it is supported by the findings in the literature that positive experiences during childhood may have decreasing effects on the risk of eating addiction, but negative experiences may increase this risk. In a study conducted by Wattick et al., the effects of childhood experiences on eating addiction in young adults were examined. The study showed that positive childhood experiences may play a reducing role in the development of eating addiction (Wattick et al. 2023). Similarly, Offer et al. examined the relationship between eating addiction and childhood traumas. They showed that eating addiction was associated with early life traumas in individuals with high body mass index (Offer et al. 2022). There are also studies in the literature examining food addiction and childhood experiences in different age groups. Guillaume et al. (2016) draw attention to the psychological differences associated with food addiction, suggesting that individuals with disordered eating behavior often show increased impulsivity and emotional distress, which are affected by childhood adversities. In addition, Hardy et al. (2018) state that food addiction shares clinical features with substance use disorders and that individuals who face significant difficulties in childhood may be more prone to both food addiction and substance abuse. Positive childhood experiences increase individuals' self‐confidence and self‐esteem. These results contribute to the development and maintenance of healthy living habits. As a result, positive childhood experiences may prevent the emergence of behavioral problems such as eating addiction by protecting both the emotional and physical health of individuals.

Another finding of our study is that an increase in the level of picky eating increases eating addiction. Research on whether an increase in the level of picky eating increases eating addiction shows that eating addiction is an important link in understanding psychiatric and medical problems (Vasiliu 2022). In addition, it has been observed that high psychological stress, depression, anxiety, and negative eating habits associated with stress (cognitive restraint, uncontrolled eating and emotional eating) are associated with high eating addiction scores (Brytek‐Matera et al. 2021). Studies on eating addiction emphasize that eating disorders (e.g., binge eating disorder) and obesity are important health problems in society (Penzenstadler et al. 2019). Furthermore, it has been reported that high‐fat and rapidly absorbed foods are particularly associated with “eating addiction” (Schulte et al. 2015). This suggests that an addiction‐like response to highly attractive foods may cause eating‐related problems such as obesity and eating disorders (Gearhardt et al. 2014). Studies show that eating addiction symptoms are more prominent in obese individuals with more severe and frequent problems such as obesity, depression, emotional eating, and eating disorders (Bourdier et al. 2020). In addition, patients who meet the criteria for eating addiction have higher levels of negative emotions, difficulties in emotion regulation, eating disorder psychopathology, and lower self‐esteem (Gearhardt et al. 2013). It is thought that picky eating habits acquired in childhood or adolescence will bring both psychiatric and medical consequences in adulthood. In the light of this information, health professionals should develop actions and policies considering that childhood experiences play an important role in improving of picky eating behaviors.

The last finding of our study was that increased Food Power Scale scores (hedonic hunger) increased eating addiction. The Food Power Scale's total score, which indicates hedonic hunger, has an important role among the factors affecting eating behavior, and high food power scores increase the risk of eating addiction (Ayyıldız et al. 2021). Studies show that there is a positive relationship between Food Power Scale scores and the desire to consume high‐energy and processed foods such as chocolate, fast‐food foods, and pasta. These foods are also reported to have the highest rate of food addiction (Erkılıç et al. 2024). High Food Power Scale scores may increase the risk of eating addiction by increasing people's cravings for unhealthy foods (Ülker et al. 2024). It is known that eating addiction is strongly associated with hedonic hunger (Gearhardt 2018). Davis et al. concluded in a study that individuals with high levels of hedonic hunger also had higher levels of eating addiction symptoms (Davis and Loxton 2014; Loxton and Tipman 2017).

Our ML and DL analyses provided nuanced insights beyond traditional regression. The SVM model achieved superior predictive accuracy (AUC = 0.92) by capturing nonlinear interactions between variables—particularly between hedonic hunger and childhood trauma—that hierarchical regression could not detect due to its linearity constraints (Figure 3). SHAP analysis (Figure 4) further revealed that hedonic hunger (Power of Food Scale) contributed 62% of the model's predictive power, aligning with neurobiological evidence implicating reward system dysregulation in addictive eating (Gearhardt et al. 2016). Notably, RF identified a subgroup with high picky eating scores but low addiction risk, suggesting potential protective factors (e.g., resilience) that warrant targeted investigation.

While hierarchical regression explained 26.6% of variance in eating addiction (Table 2), ML/DL models improved predictive performance (AUC = 0.92) by modeling complex feature interactions. For example, regression identified Negative Childhood Experiences as a significant predictor (*β* = 0.36, *p* < 0.001), but SHAP values indicated this effect was amplified in participants with concurrent high hedonic hunger (≥ 75th percentile). Conversely, Positive Childhood Experiences showed no effect in regression (*p* = 0.57) but appeared protective in RF splits for low‐risk subgroups. This divergence underscores ML's ability to uncover subgroup‐specific patterns masked by linear models. In the literature, hedonic hunger is reported to have a strong and positive relationship with as a result, similar to the literature, it was revealed in this study that hedonic hunger is an important factor in eating addiction.

## 5 Conclusion

This study revealed that negative childhood experiences, picky eating behaviors, and hedonic hunger significantly predict eating addiction in university students, with ML models (particularly SVM with AUC = 0.92) outperforming traditional regression by identifying high‐risk subgroups through nonlinear interactions. The findings underscore the importance of early interventions targeting reward dysregulation in trauma‐exposed individuals and suggest the need for comprehensive screening tools combining psychological and behavioral markers. These results have important clinical implications, suggesting that: (1) screening programs for university students should incorporate assessments of both childhood adversity and current hedonic hunger, (2) interventions targeting reward system sensitivity may be particularly effective for trauma‐exposed individuals, and (3) picky eating behaviors may serve as an early warning sign for developing addiction‐like eating patterns. Future research directions should include longitudinal studies to establish temporal relationships using recurrent neural networks, investigation of protective factors in resilient subgroups identified by RF analysis, cross‐cultural validation in more diverse populations (particularly with greater male representation beyond our 19.4% sample), and integration of neuroimaging biomarkers to complement self‐report measures. In addition, while ML models showed excellent predictive utility, further work is needed to develop explainable AI approaches that can translate these complex algorithms into clinically actionable tools.

## 6 Strengths and Limitations

This study has several strengths. First, the use of both traditional regression and ML approaches provided a comprehensive understanding of predictors for eating addiction, with ML models uncovering nonlinear interactions missed by linear analyses. Second, the large sample size (*n* = 681) enhanced statistical power, and the inclusion of validated scales (e.g., Yale Food Addiction Scale) improved measurement reliability. Third, SHAP analysis offered interpretable insights into feature importance, bridging the gap between predictive accuracy and clinical utility.

However, some limitations should be acknowledged. The cross‐sectional design precludes causal inferences, and self‐reported data may be subject to recall bias. Although ML models performed well, their “black‐box” nature limits immediate clinical translation. In addition, the sample was predominantly female (80.6%) and from a single Turkish university, which may affect generalizability. Future longitudinal studies with diverse populations could address these issues.

## Author Contributions

**Muhammet Ali Aydin**: formal analysis, methodology, writing–review and editing, validation. **Ceren Karabulutlu**: data curation, writing–original draft, software. **Izzet Ulker**: conceptualization, methodology, writing–original draft, writing–review and editing. **Metin Yildiz**: conceptualization, formal analysis, writing–review and editing, supervision.

## Ethics Statement

Before starting the study, written permission was obtained from Erzurum Technical University Scientific Research and Publication Ethics Committee (Date: 04.04.2024 04:04) and the relevant university where the research would be conducted. The first question of the online questionnaire form was prepared as “Do you agree to participate in the study?” and permission was obtained from the students in this way. The Declaration of Helsinki was followed within the scope of the study.

## Consent

Informed consent was obtained from all subjects involved in the study.

## Conflicts of Interest

The authors declare no conflicts of interest.

## Peer Review

The peer review history for this article is available at https://publons.com/publon/10.1002/brb3.70667

References

## Data Availability

Data will be made available on request.
